# Value of Breast Cancer Molecular Subtypes and Ki67 Expression for the Prediction of Efficacy and Prognosis of Neoadjuvant Chemotherapy in a Chinese Population

**DOI:** 10.1097/MD.0000000000003518

**Published:** 2016-05-06

**Authors:** Jiayu Wang, Die Sang, Binghe Xu, Peng Yuan, Fei Ma, Yang Luo, Qing Li, Pin Zhang, Ruigang Cai, Ying Fan, Shanshan Chen, Qiao Li

**Affiliations:** From the Department of Medical Oncology, Cancer Hospital, Chinese Academy of Medical Sciences & Peking Union Medical College, Panjiayuannanli, Chaoyang District, Beijing, China.

## Abstract

The aim of the study was to determine the predictive role of breast cancer subtypes in the efficacy and prognosis of neoadjuvant chemotherapy (NCT) regimens combining taxanes and anthracyclines.

Data from 240 patients with breast cancer who received surgery after 4 to 6 weeks of NCT were retrospectively analyzed. The patients were classified into luminal A, luminal B, HER2 overexpression, and triple negative breast cancer (TNBC) as well as low Ki67 (≤ 14%) and high Ki67 (> 14%) expression groups using immunohistochemistry. NCT outcome parameters were pathological complete response (pCR), clinical complete response (CR), partial response (PR), stable disease (SD), and progressive disease (PD) 4 weeks after surgery. Long-term outcome parameters were disease-free survival (DFS) with a follow-up time of 3 to 56 months.

pCR rates were 1.6%, 13.4%, 22.6%, and 23.8% in patients with luminal A, luminal B, HER2, and TNBC cancers, respectively. High pCR rates correlated with high Ki67 expression (> 40%) (*P* < 0.001, HR = 0.17, 95% CI: 0.074–0.37) and negative estrogen receptor (ER) status (*P* < 0.001, HR = 3.74, 95% CI: 1.71–8.12) in a multivariate analysis. However, the DFS rate of luminal A breast cancer was the highest compared to all other groups, but only significantly higher compared to luminal B (*P* = 0.035, HR = 1.480, 95% CI: 1.060–1.967) patients and correlated with Ki67 expression > 40% (*P* = 0.005).

Luminal A type patients derived the least benefit from neoadjuvant chemotherapy but had better long-term prognoses. ER status and Ki67 expression served as efficacy predictors for NCT, whereas only Ki67 expression > 40% correlated with long-term treatment outcomes.

## INTRODUCTION

Breast cancer accounted for ∼21% of all cancer cases worldwide from 1995 to 2009,^1,2^ and subsequent studies have noted a rapid increase in the incidence of breast cancer in China,^1,3,4^ especially among women 20 to 45 years of age. Thus, breast cancer has become one of the most common types of malignancy in Chinese women.^3,5^ The earliest classification into estrogen and progesterone receptor positive and negative breast cancers has been extended to the human epidermal growth factor receptor 2 (HER2) expressing types. The 2011 and 2013 St. Gallen Consensus Conference added Ki-67 for the determination of proliferation rates to the markers for breast cancer subtype categorization into luminal A, luminal B, as well as triple negative basal-like (ER neg, PgR neg, and HER2 neg) and HER2 overexpressing types.^6,7^ These breast cancer molecular subtypes have been proposed to serve as risk factor and prognosis indicators, but their role in evaluating risk and prognosis of an individual patient is limited.^8,9^ In addition, threshold values for defining high and low Ki-67 expression are not clearly defined and vary between laboratories.^7,10,11^ However, particularly for endocrine therapies, a classification of breast tumors is crucial, so avoiding the unnecessary burden of ineffective therapies for patients with resistant tumors.^12^ Neoadjuvant chemotherapy (NCT), initially adopted to downgrade inoperable cancers into operable cancers, became a well-accepted treatment option for breast cancer to minimize tumor size^13^ and to evaluate the efficiency of a regime with respect to micrometastases^14^ as well as for adjuvant chemotherapy medication adjustments.^15^ It has been shown that neoadjuvant therapy of breast cancer was equivalent to adjuvant therapy regarding survival and the overall disease progression,^16^ but it has been suggested that patients reaching pCR after neoadjuvant chemotherapy have favorable outcomes.^17^ However, whether pCR after neoadjuvant breast cancer therapy can serve as a surrogate endpoint marker for long-term outcomes is still under debate.^18^ In the present study, we analyzed molecular breast cancer subtypes related pathologically complete response (pCR) and disease-free survival (DFS) outcomes after neoadjuvant chemotherapies in a Chinese cohort of patients in order to identify specific efficacy predictors and thus improve individualized treatment of breast cancer.

## PATIENTS AND METHODS

### Patients

In a retrospective study, we included 240 female breast cancer patients without metastasis who were admitted to our hospital between January 2009 and January 2014. The median age was 48 years (23–73 years), the clinical stage was II or III, the Eastern Cooperative Oncology Group (ECOG) scores ranged from 0 to 1, and 98% of patients were diagnosed with invasive ductal carcinoma according to pathological examinations after core needle biopsies. The research protocol was approved by the medical ethical committee of the Cancer Hospital of the Chinese Academy of Medical Sciences and informed consent was obtained from all participants.

### Medications and Surgery

#### Neoadjuvant Medications

Chemotherapy doses were epirubicin 75 mg/m^2^ IV day 1, paclitaxel 175 mg/m^2^ IV day 2, or docetaxel 75 mg/m^2^ IV day 2 in 21 days for 1 cycle. A total of 160 patients received epirubicin + paclitaxel and 80 patients received epirubicin + docetaxel.

#### Adjuvant Medication

Postoperative adjuvant chemotherapies (6–8 cycles) were administered to 166 patients, out of which the regimens changed for 67 patients. Also, 21 HER2-positive patients received postoperative trastuzumab for 1 year and 10 patients were treated with paclitaxel + platinum-based drugs + trastuzumab after operation. However, 170 patients who were positive for the estrogen receptor were treated with adjuvant endocrine therapy.

All patients were operated within 1 month after the end of NCT (2–6 cycles, median 6 cycles, depending on their responses, but in the case of PD after 2 cycles, NCT was discontinued) and the interventions were modified radical mastectomy for 223 and breast-conserving operations for 23 women. Also, 180 patients received postoperative radiotherapy.

### Efficacy Evaluation

#### NCT Outcomes 4 Weeks After Surgery

RECIST version 1.1^19^ was used to assess the treatment response. NCT outcome parameters were a pathologically complete response (pCR), clinical complete response (CR), partial response (PR), stable disease (SD), and progressive disease (PD), 4 weeks after surgery. pCR was defined as no histological evidence of malignancies or only in situ residuals in breast tissue after surgery, and complete disappearance of lymph node metastasis. CR was defined as disappearance of all known lesions for >4 weeks. PR was defined as at least a 30% decrease in the sum of the largest diameters of target lesions for >4 weeks. PD was defined as at least a 20% increase in the sum of the largest diameters of target lesions or new lesions detected. SD was defined as a reduction in the largest sum diameters of tumors by no more than 30% or an increase of no more than 20% for 4 weeks.

#### Long-Term Outcomes With a Follow-Up Time of 3 to 56 Months (Median 29 Months)

Disease-free survival (DFS) refers to the time from start of NCTs to the appearance of local recurrence, regional metastasis, second primary cancer, distant metastasis, or death.

#### Breast Cancer Classification

Breast cancer staging was performed according to the American Joint Committee on Cancer (AJCC) TNM system.^20^ The tumors were categorized into luminal A and luminal B, as well as HER2 and triple negative types according to Goldhirsch et al^6^ (Table 1).

**TABLE 1:**
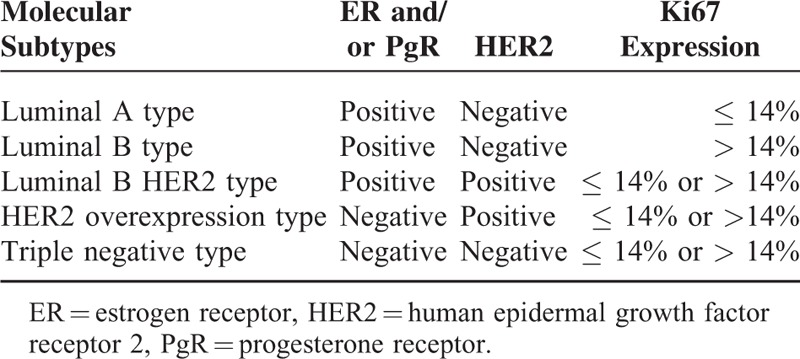
Molecular Breast Cancer Subtypes Based on Immunohistochemistry

Estrogen receptor (ER) and progesterone receptor (PgR) positivity was conservatively defined to those cells with tumor nuclear staining of > 10%.^21,22^ A positive HER2 result was defined as a +++ staining of > 30% of invasive tumor cells and a fluorescent in situ hybridization (FISH) result of > 6 HER2 gene copies per nucleus; a negative result was defined as an immunohistochemistry (IHC) staining of 0 or + and a FISH result of < 4 HER2 gene copies per nucleus.^23^ To identify Ki67-positive tumor cells we used the method described by Bukholm et al.^24^ Briefly, 10 fields of cell nuclei Ki67-stained cells (pale yellow or brownish yellow) were randomly chosen and 500 cells were counted under each field. Then, the percentages of Ki67-positive cells were calculated. Ki67 ≤ 14% was defined as low expression and Ki67 > 14% as high expression^6,25^ for the classifications, whereas for pCR and DFS correlation estimates different expression percentages were used for the calculations in order to evaluate the best suitable cut-off value.

### Statistical Analysis

SPSS19.0 software (SPSS Statistics for Windows, Version 19.0. Armonk, NY: IBM Corp.) was used for all calculations. A χ^2^ test was used to analyze the relationship between molecular subtypes and clinicopathological features and for univariate analysis of clinicopathological indicators and pCR. The Kaplan–Meier method was used for survival analysis and a bivariate logistic regression model for multivariate analysis. A Cox multivariate regression model was used to determine and analyze risk factors effecting prognosis (DFS). *P* < 0.05 was considered to be statistically significant.

## RESULTS

Among the 240 breast cancer patients, there were 61 (25.4%) with luminal A, 127 (52.9%) with luminal B type, 31 (12.6%) with HER2 overexpression, and 21 (8.8%) were triple negative (TN) types. As shown in Table 2, age and N stage before chemotherapy did not correlate with molecular subtypes, whereas menstrual status (*P* = 0.026), T stage before chemotherapy (*P* = 0.004), and Ki67 expressions with a cut-off threshold of 14% (*P* < 0.001) were significantly differently distributed between the molecular subtypes, being the highest in luminal B type cancers. The response rates to neoadjuvant chemotherapies (pCR vs non-pCR) also significantly differed (*P* = 0.007) between the subgroups (Table 2).

**TABLE 2:**
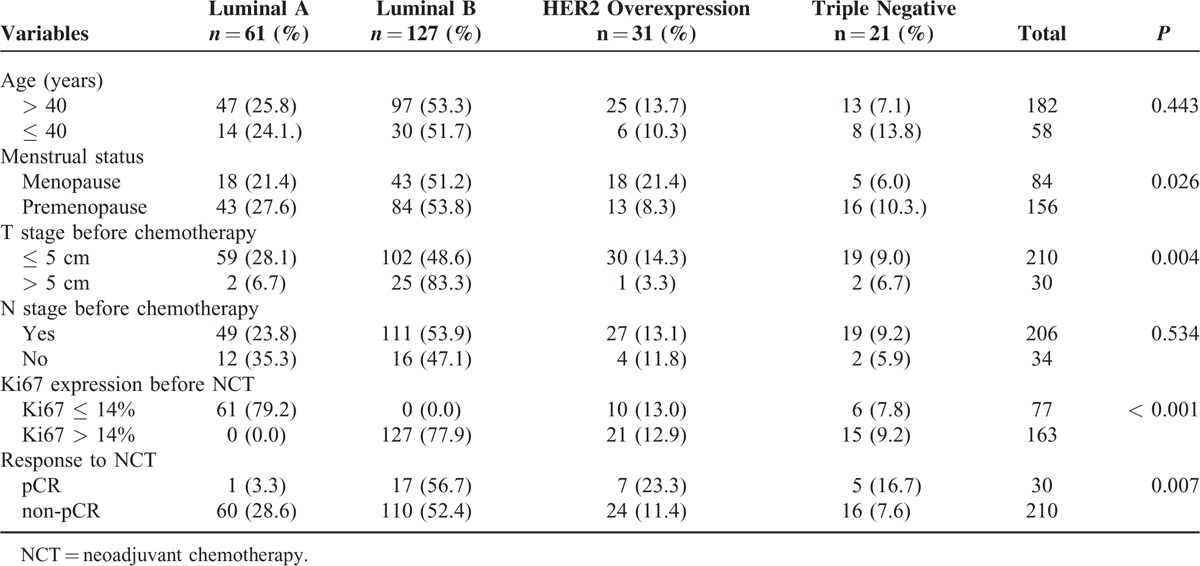
Correlation Between Molecular Subtypes and Clinicopathological Features (Case Numbers [%])

According to the pathological response after using taxanes in combination with anthracyclines, 96.3% (231/240) of patients reached PR + CR + SD, 3.7% (9/240) reached PD, and 30 patients (12.5%) achieved pCR, whereas 53 (22.1%) of patients achieved pCR in breast lesions. According to a univariate analysis of clinicopathological indicators and pCR, ER (*P* < 0.001) and Ki67 (*P* < 0.001) statuses correlated significantly with pCR, with the most significant percentage cut-off value for Ki67 expression being 40% (Table 3). pCR rates were higher in ER-negative than in ER-positive patients (23.2% vs 7.0%, *P* < 0.001) and in patients with Ki67 > 40% compared to those with Ki67 ≤ 40% (33.3% vs 8.1%, *P* < 0.001). No significant correlation was found between pCR and age, size of breast tumors, PR, menstrual status, and condition of axillary lymph nodes before chemotherapy or HER2 overexpression (Table 3).

**TABLE 3:**
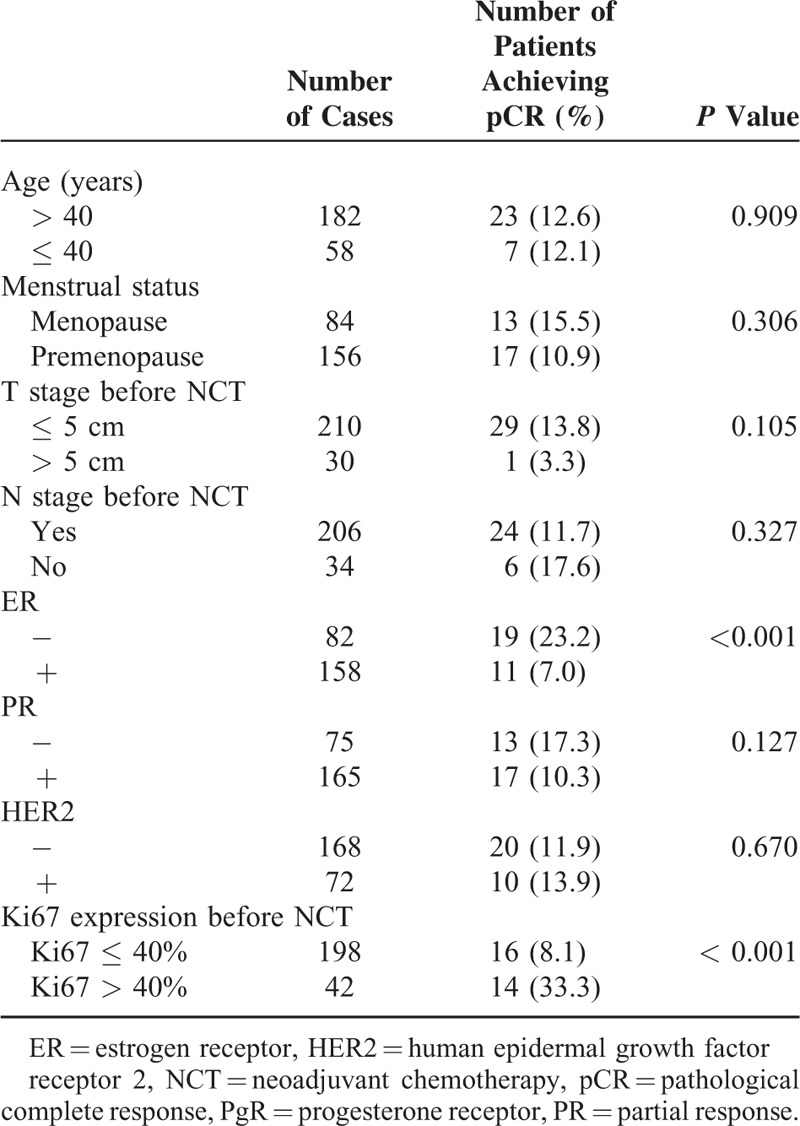
Univariate Analysis of Clinical Indicators and pCR

Also the results of a bivariate logistic regression analysis of the correlations between pCR and clinical stages, molecular subtypes, ER status and Ki67 index before chemotherapy, showed significant correlations for ER (*P* = 0.014, HR = 3.341, 95% CI: 1.280–8.724) as well as Ki67 index (Ki67 of 40% as the threshold level, *P* < 0.001, HR = 0.189, 95% CI: 0.079–0.448) and pCR rates.

**FIGURE 1:**
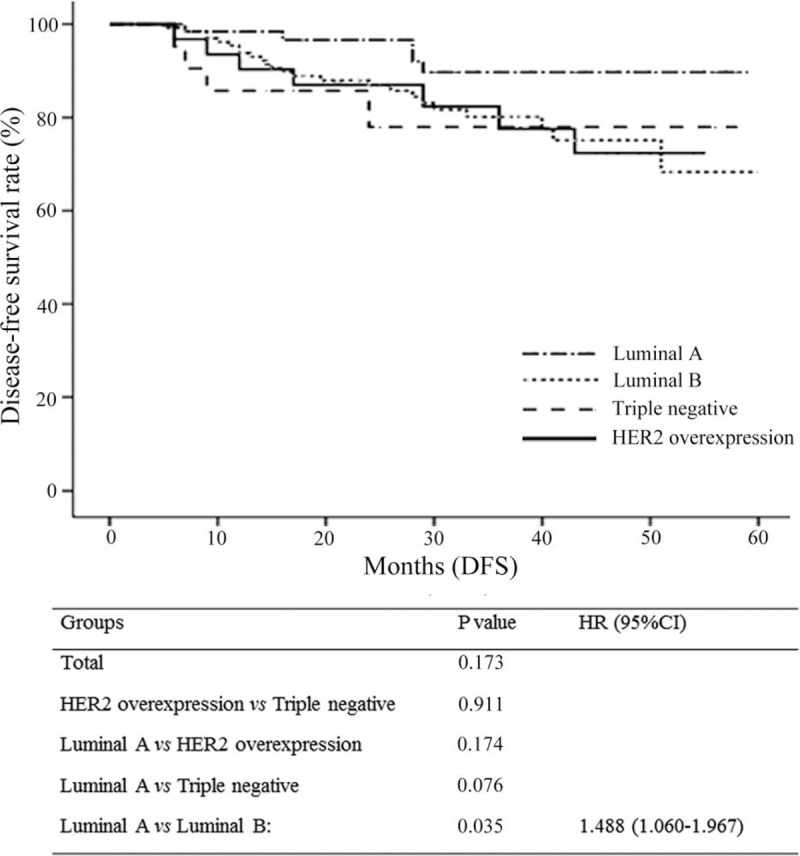
Comparison of disease-free survival rates among the indicated breast cancer groups.

Luminal A type patients had the lowest pCR and response rates to chemotherapy, followed by luminal B type patients. The pCR rate was the highest in patients with HER2 expression followed by TNBC patients (Table 4). The 240 patients were followed for 3 to 56 months, with a median follow-up time of 29 months. Until May 2014 as the last follow-up, 26 patients had recurrent and metastatic lesions and 14 patients died. DFS rates were significantly superior (*P* = 0.035) in patients with luminal A type than in those with luminal B type breast cancer, with a median DFS of 35 and 26 months, respectively (Figure 1).

**TABLE 4:**
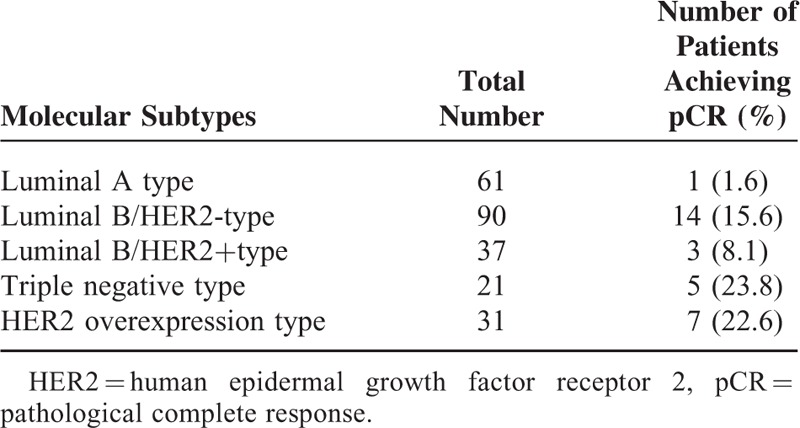
Molecular Subtypes and pCR

A Cox regression model was used to analyze factors affecting DFS, including age, menstrual status, Ki67 expression, ER, PR, HER2, lymph node status before chemotherapy, and molecular subtypes. Only the Ki67 expression level before chemotherapy was an independent prognostic factor, with a significantly higher DFS rate in patients with a Ki67 expression ≤ 40% compared to those with > 40% before NCT (*P* = 0.005).

## DISCUSSION

In our study, we analyzed the efficacy difference of neoadjuvant chemotherapy regimens in 240 breast cancer patients and found that patients with luminal A (1.6%) and luminal B (13.4%) types had the lowest pCR rates followed by HER2 overexpressing (22.6%) and triple negative (23.8%) types (Table 4). This is in agreement with previous reports, which noted that triple negative and HER2+ subtypes were more sensitive to anthracycline-based neoadjuvant chemotherapies than luminal breast cancers.^26,27^ Also, others reported that the response to neoadjuvant chemotherapy was significantly higher in patients with endocrine nonresponsive tumors,^28^ with pCR rates of 24% in hormone receptor (HR)-negative and 8% in HR-positive tumors.^29^ Patients with luminal B/HER2-subtypes had a higher pCR rate than those with the luminal B/ HER2+ subtypes (15.6% and 8.1%, respectively), which is in accordance with a recent report in which 25% of patients with luminal B/HER2- and only 8% with luminal B/ HER2+ subtypes achieved pCR after neoadjuvant chemotherapies.^30^ Interestingly, with neoadjuvant medication achieved pCR in luminal B/HER2-, HER2 overexpressing and triple negative subtypes, which showed the highest pCR rates in our study, but not for luminal A and luminal B/HER2+ subtypes, which showed the lowest pCR rates, has been proposed as a surrogate end point for favorable prognosis in a previous meta-analysis.^31^ On the other hand, a meta-regression analysis of 29 randomized prospective studies revealed that pCR is not a surrogate end point for outcomes in patients with breast cancer.^32^ In our DFS analysis, luminal A subtype patients had the lowest pCR rates and the best prognosis (Figure 1). Luminal A type breast cancer is the most common and least aggressive type with the lowest mortality rate.^33^ In addition, luminal A mortality rates were reported to be constant over time with mortality rates of luminal B HER2-positive and nonluminal subtypes tending to peak within 5 years after diagnosis which then declined over time,^34^ findings also reflected in our data of significantly better DFS rates of luminal A versus luminal B type patients (*P* = 0.035). The Ki67 index, which indicates the cell proliferation rate, has been the focus of various studies and is recognized as a prognostic predictor for breast cancer.^35^ Patients with a high expression of Ki67 are more sensitive to chemotherapy, have higher pCR rates, and benefit more from chemotherapy compared to those with a low Ki67 expression.^36^ In a study conducted by Ohno et al, 477 patients with locally advanced breast cancer were given 4 weeks of neoadjuvant chemotherapy with fluorouracil + epirubicin + cyclophosphamide (FEC) and then randomized into docetaxel + capecitabine (TX) or docetaxel monotherapy (T) groups. They found that the pCR rate was higher in patients with a high Ki67 expression (Ki67 > 10%) than in patients with a low expression (Ki67 ≤ 10%) (12.3% vs 6.5%, *P* = 0.0004).^37^ Also in our study, patients with Ki67 > 40% were more sensitive to chemotherapy and had significantly higher pCR rates compared with lower Ki67 ≤ 40% expressing patients (33.3% vs 8.1%, *P* < 0.001). In addition, Ki67 expression < 40% was a marker for favorable DFS rates, which is in agreement with a previous study in which patients with Ki-67 values > 45 % had reduced DFS.^38^ A limitation of our study was the relatively short follow-up period of 3 to 56 months (median 29 months), which did not cover the different recurrence patterns of the breast cancer molecular subtypes, and that our study was retrospective.

In conclusion, patients with different types of breast cancer had different responses to NCT regimens. High Ki67 expression and ER status were factors determining neoadjuvant chemotherapy pCR outcomes, whereas only Ki67 expression significantly correlated with DFS rates. However, though luminal A cancer patients had the lowest pCR rate after NCT, they had the highest DFS rate, which was significantly superior to that of luminal B patients. The evaluation of the role of molecular subtype for prognosis of breast cancer NCT regimens needs further long-term studies with more patient-orientated NCT regimens.
